# Brain volumetric and microstructural correlates of executive and motor performance in aged rhesus monkeys

**DOI:** 10.3389/fnagi.2012.00031

**Published:** 2012-11-08

**Authors:** Aadhavi Sridharan, Auriel A. Willette, Barbara B. Bendlin, Andrew L. Alexander, Christopher L. Coe, Mary L. Voytko, Ricki J. Colman, Joseph W. Kemnitz, Richard H. Weindruch, Sterling C. Johnson

**Affiliations:** ^1^Neuroscience Training Program, University of Wisconsin-MadisonMadison, WI, USA; ^2^Medical Scientist Training Program, University of Wisconsin-MadisonMadison, WI, USA; ^3^Geriatric Research Education and Clinical Center, Wm. S. Middleton Memorial Veterans HospitalMadison, WI, USA; ^4^Wisconsin Alzheimer's Disease Research Center, University of Wisconsin School of Medicine and Public HealthMadison, WI, USA; ^5^Department of Medicine, University of Wisconsin-MadisonMadison, WI, USA; ^6^Waisman Laboratory for Brain Imaging and Behavior, University of Wisconsin-MadisonMadison, WI, USA; ^7^Department of Psychology, University of Wisconsin-MadisonMadison, WI, USA; ^8^Department of Neurobiology and Anatomy, Wake Forest School of MedicineWinston-Salem, NC, USA; ^9^Wisconsin National Primate Research CenterMadison, WI, USA; ^10^Department of Cell and Regenerative Biology, University of Wisconsin School of Medicine and Public HealthMadison, WI, USA; ^11^Institute for Clinical and Translational Research, University of Wisconsin-MadisonMadison, WI, USA

**Keywords:** aging, macaque, cognition, magnetic resonance imaging, motor

## Abstract

The aged rhesus macaque exhibits brain atrophy and behavioral deficits similar to normal aging in humans. Here we studied the association between cognitive and motor performance and anatomic and microstructural brain integrity measured with 3T magnetic resonance imaging in aged monkeys. About half of these animals were maintained on moderate calorie restriction (CR), the only intervention shown to delay the aging process in lower animals. T1-weighted anatomic and diffusion tensor images were used to obtain gray matter (GM) volume and fractional anisotropy (FA) and mean diffusivity (MD), respectively. We tested the extent to which brain health indexed by GM volume, FA, and MD were related to executive and motor function, and determined the effect of the dietary intervention on this relationship. We hypothesized that fewer errors on the executive function test and faster motor response times would be correlated with higher volume, higher FA, and lower MD in frontal areas that mediate executive function, and in motor, premotor, subcortical, and cerebellar areas underlying goal-directed motor behaviors. Higher error percentage on a cognitive conceptual shift task was significantly associated with lower GM volume in frontal and parietal cortices, and lower FA in major association fiber bundles. Similarly, slower performance time on the motor task was significantly correlated with lower volumetric measures in cortical, subcortical, and cerebellar areas and decreased FA in several major association fiber bundles. Notably, performance during the acquisition phase of the hardest level of the motor task was significantly associated with anterior mesial temporal lobe volume. Finally, these brain-behavior correlations for the motor task were attenuated in CR animals compared to controls, indicating a potential protective effect of the dietary intervention.

## Introduction

Aging in humans results in both gray matter (GM) and white matter (WM) brain atrophy, and a decline in microstructural integrity, as measured in postmortem studies and studies using non-invasive magnetic resonance imaging (MRI) techniques (Raz et al., 1997, 2004; Gunning-Dixon and Raz, 2000; Esiri, 2007; Bendlin et al., 2010). These gross anatomical and microstructural alterations are associated with cognitive and behavioral changes, including a decline in performance on tests of working memory and executive and motor function (Gunning-Dixon and Raz, 2000; Bendlin et al., 2010), although the relationships between age-related brain changes and cognitive function are still incompletely known.

The rhesus monkey (*Macaca mulatta*) provides a valuable animal model for studying human aging because, like humans, it exhibits age-related brain atrophy (albeit to a lesser degree) with selective cognitive deficits. There is a decrease in total parenchymal volume in female rhesus monkeys as a function of age, attributed exclusively to GM loss prior to 15 years of age and mostly to WM loss in later years (Andersen et al., 1999). Younger rhesus monkeys have larger caudate and putamen volumes than middle-aged and old monkeys (Matochik et al., 2000), and there is an overall decrease in forebrain GM and WM, caudate and globus pallidus volumes from young to old age (Wisco et al., 2008). In addition, there is a significant age-related decrease in WM integrity in the superior longitudinal fasciculus (SLF) II, cingulum bundle, as well as corpus callosum (Makris et al., 2007). Concurrently, these monkeys also show evidence of progressive cognitive decline as early as in middle age. Middle-aged and old monkeys demonstrate significant impairments on executive function tests that assess abstraction, concept formation, set shifting, and show increased perseverative tendencies (Lai et al., 1995; Moore et al., 2003, 2006). This shift is attributed to an age-related decline in prefrontal cortex function as well as to the age-related decline in WM integrity (Makris et al., 2007; Moore et al., 2009). While young males outperform young females on a spatial memory task, aged males do not perform significantly better than old females (Lacreuse et al., 1999). Old monkeys also display robust learning deficits and significantly impaired memory compared to younger animals (Moss et al., 1997; Shamy et al., 2006). Finally, younger animals show faster motor learning than aged animals, and increasing age is significantly correlated with slower performance times on fine motor hand tasks (Zhang et al., 2000; Moore et al., 2010).

Although several studies have evaluated structural and behavioral changes related to aging in this non-human primate model, only a few have examined the relationship between anatomical changes and behavioral alterations (Shamy et al., 2006, 2011; Makris et al., 2007; Alexander et al., 2008; Wisco et al., 2008). Makris et al. (2007) studied a cohort of young (6.5–15 years of age) and aged (19.5–30.5 years of age) rhesus monkeys using diffusion tensor imaging (DTI) and behavioral testing. Using an *a priori* region of interest (ROI) approach, they demonstrated a linear relationship between regional WM integrity in frontal association pathways, such as the SLF II and cingulum bundle, and executive function. Wisco et al. (2008) assessed brain volume in young (5–12 years), middle-aged (16–19 years), and old (24–30 years) animals and demonstrated an age-related decline in forebrain volume. However, forebrain volumetric measures were not related to learning and memory performance. As the authors noted, this lack of brain-behavior correlation may have been due to a broad inclusion of anatomic areas in the forebrain ROI volume measurement. Shamy and colleagues (2011) demonstrated that age was inversely correlated with striatal, dorsolateral prefrontal, and anterior cingulate cortex ROI volumes and that hippocampal ROI volume predicted spatiotemporal memory task acquisition, striatal volume predicted recognition memory task acquisition, and prefrontal volume predicted accuracy on recognition memory task in aged rhesus monkeys. While these ROI studies are a first step in understanding the relationship between cognitive function and brain health, additional work is clearly needed. Spatial precision may be improved with voxel level analyses, and the influence of age on brain-behavioral relationships may be evident on tasks with robust age effects in the human such as speeded fine motor coordination, and fluid cognitive processes such as concept formation and reasoning.

The rhesus macaque is also ideal for studying dietary and drug interventions that might preserve brain health and slow cognitive decline during aging. One powerful intervention consistently shown to delay the process of aging and prolong lifespan in animal models, including yeast, worms, flies, fish, and rodents is moderate calorie restriction (CR). Recent work indicates that CR is beneficial in delaying onset of age-related diseases and may potentially increase lifespan in non-human primates (Colman et al., 2009; Kemnitz, 2011). In rhesus monkeys, CR results in the preservation of GM volume in the midcingulate cortex, bilateral lateral temporal cortex, and right dorosolateral frontal cortex, and WM integrity in the fronto-occipital fasciculus, SLF, external capsule, and brainstem (Colman et al., 2009; Bendlin et al., 2011). Age-related iron accumulation in the basal ganglia, red nucleus, and parietal, temporal, and perirhinal cortex is also attenuated in monkeys consuming a CR diet (Kastman et al., 2010). Despite these intriguing findings, little work has been done to assess the functional relevance of this protective effect of CR on brain health.

In the present study, our primary aim was to investigate brain-behavior associations between MRI-derived anatomic and microstructural brain health measures and cognitive and motor performance in an aged cohort of rhesus macaque (19–29 years of age) using a cross-sectional design. Our secondary aim was to determine the benefits of CR, an intervention believed to slow the process of aging, on these brain-behavior relationships. T1-weighted volumetric imaging was used to derive GM volume. DTI, which is sensitive to Brownian displacement of water molecules, was used to investigate microscopic tissue integrity (Basser, 1995; Basser and Pierpaoli, 1996). Two measures derived from DTI were used: fractional anisotropy (FA), a measure of directionality of diffusion, and mean diffusivity (MD), a measure of the overall displacement of water. Performances on a modified version of Wisconsin Card Sorting Task, which is widely used in humans and is sensitive to prefrontal cortex dysfunction (Moore et al., 2006), and on monkey movement assessment panel (mMAP) (Gash et al., 1999) were used for behavioral assessment. We hypothesized that GM volume, WM FA, and MD measures would predict behavioral performance, and that CR would have a protective effect on these associations. Studies in humans show that GM volume and FA decline with age, whereas MD increases with age (Raz et al., 1997, 1998; Pfefferbaum et al., 2000; Pfefferbaum and Sullivan, 2003). Therefore, higher GM volume and greater FA (and lower MD), specifically in frontal areas that mediate executive function, and in motor, premotor, and subcortical basal nuclei that underlie goal-directed motor behaviors, were hypothesized to be related to better respective task performance across animals, with monkeys on a CR diet expected to show greater preservation of brain health and superior behavioral performance. However, because both cognitive and motor function involve a distributed network, we used a voxel-wise approach which facilitates analysis across the whole brain without being restricted to a limited number of regions of interest.

## Materials and methods

### Animals

All animals used in these analyses are part of the longitudinal Dietary Restriction and Aging Study at the Wisconsin National Primate Research Center (WNPRC; Table 1). Monkeys (*Macaca mulatta*), aged 19–29 years, were either fed *ad libitum* (controls) or maintained on a moderately restricted diet (30% reduced intake from their individual baseline), with both groups receiving equivalent diet supplements. The CR diet was initiated in young adulthood and the length of CR diet ranged from 12 to 17 years. Further details of the dietary manipulation and experimental setup have been described extensively elsewhere (Kemnitz et al., 1993; Ramsey et al., 2000). The study was approved by the Institutional Animal Care and Use Committee at the University of Madison-Wisconsin.

**Table 1:** **Animal demographics**.

	**Controls**	**CR**
**COGNITION ANALYSES**		
*N*	3	12
Age (years)	22.24 ± 2.29	23.54 ± 0.78
Gender (M/F)	3/0	7/5
%PE	53.31 ± 8.54	57.19 ± 3.74
%TE	50.61 ± 1.75	50.12 ± 0.77
**MOTOR ANALYSES**		
*N*	16	11
Age (years)	24.35 ± 0.64	22.76 ± 0.95
Gender (M/F)	9/7	7/4
Reaction time (s)	0.24 ± 0.01	0.26 ± 0.01
Coarse movement time (s)	0.48 ± 0.02	0.51 ± 0.02

Notes: Data represent mean ±SEM. %PE, total perseverative errors as a percentage of total number of errors. %TE, total errors as a percentage of total number of trials.

### Cognition task: Wisconsin Card Sort Test (WCST)

Monkeys were behaviorally trained and tested in a computer-controlled touch screen testing apparatus, similar to an apparatus described previously (Voytko et al., 2008, 2009). In this system, monkeys were located in a transport cage positioned in front of a 17″ touch screen monitor in a sound-attenuated cubicle. The touch screen was interfaced with a computer that controlled all stimulus and reward presentations, and data collection. A reward of low-calorie banana pellets was used, dispensed into a centrally located food cup. The WCST task is similar to the one developed by Moss and colleagues (Moore et al., 2002, 2003) and assesses abstract reasoning, concept formation, and ability to make conceptual shifts in response to changing task demands. Three stimuli appeared in pseudo-random locations on the monitor on each trial. The stimuli differed in color (red, green, blue) and shape (triangle, star, circle). An initial category exemplar (red) was tested for acquisition followed by the testing of three category shifts (triangle, blue, star). For the initial acquisition stage, the monkey was required to touch the red stimulus regardless of its shape on ten consecutive trials to reach criterion. At that time, the reward contingency was switched so that reward was provided only when the triangle, regardless of its color, was touched for ten consecutive trials. The contingency was switched again so that reward was provided only when the blue stimulus, regardless of its shape, was touched for ten consecutive trials. The final contingency condition required touching the star shape, regardless of its color. A 15-s inter-trial interval was used and daily test sessions consisted of 80 trials. All possible combinations of stimuli appeared on the monitor over a four-day cycle and were repeated until criterion was achieved at each stage. We recorded the total number of trials and errors to achieve criterion for each category and total number of perseverative errors (i.e., choosing a stimulus containing a component of the previous rewarded category). Total perseverative errors as a percentage of total number of errors (%PE), and total errors as a percentage of total trials (%TE) were used for correlational analyses. For each animal, the time from the start of testing until the animal made the first choice (response latency) for each day of testing was also recorded to assess motivational differences between groups.

### Motor task: monkey movement assessment panel (mMAP)

The monkey movement assessment panel (mMAP) is an automated system for testing coarse and fine upper extremity motor function (Gash et al., 1999). The details of this method of fine and coarse motor assessment have been described previously (Kastman et al., 2010). The device consists of an outer and inner chamber with openings for the subject to pass its arm through. The inner chamber consists of a receptacle with a platform, straight rod, or curved rod, with each higher level representing increasing level of task difficulty to obtain the reward. The trial begins when the food stimulus is placed in the receptacle. The animal must retrieve the food off receptacle, and withdraw its arm back into the home cage to complete the trial. The time spent with the arm in the outer chamber (coarse movement time) and the duration in the inner chamber removing the reward (fine movement time) were automatically recorded by photodiodes attached to the device and logged by a computer attached to the apparatus. There were 12 trials (6 for each hand) in a given day with a 30 s delay between trials. Each task level was tested for 10 days before proceeding to the next level. Because motor learning is more evident in complex tasks (Gash et al., 1999), fine motor performance for the hardest task, the curved rod, was further divided into two phases: an acquisition phase when the animals were learning the task and a proficiency phase when performance reached an asymptote. The two phases were determined using statistical techniques previously described (Kastman et al., 2010).

### MRI acquisition

Image acquisition parameters have been reported previously (McLaren et al., 2009; Bendlin et al., 2011). Animals were anesthetized after an overnight fast and scanned in the morning on a General Electric 3.0 T Signa MR Unit (GE Medical Systems, Milwaukee, WI, USA) with a quadrature transmit/receive volume coil (18 cm diameter) at the Waisman Center for Brain Imaging and Behavior. Prior to the scanning procedure, monkeys were given ketamine (up to 15 mg/kg intramuscular, IM) or alternative anesthesia in consultation with a WNPRC veterinarian and xylazine (up to 0.6 mg/kg, IM) prior to the scan. Subjects were occasionally given a booster injection of ketamine (7–15 mg/kg, IM or intravascular, IV) with or without xylazine (0.2–0.6 mg/kg, IM or IV) during the scan to maintain the plane of anesthesia. T1-weighted volumetric scan parameters include: TR = 8.772 ms, TE = 1.876 ms, inversion time = 600 ms, flip angle = 10°, NEX = 2, FOV = 160 mm, matrix = 256 × 224, section thickness = 0.7 mm, no gap. DTI was performed in the axial plane using a single-shot, spin-echo, diffusion-weighted echo-planar imaging sequence with diffusion gradients in 12 optimal directions. DTI parameters include: *b* = 816 s/mm^2^, TR = 10000 ms, TE = 77.2 ms, NEX = 6, FOV = 160 mm, matrix = 120 × 120, section thickness = 2.5 mm, no gap. A higher-order shimming protocol was run before the DTI scan to minimize image distortion. Voxels were resampled to 0.5 × 0.5 × 0.5 mm for T1-weighted and DTI sequences. Animals also underwent other scans including a T2-weighted scan, which was used to identify potential brain lesions.

Forty-eight animals underwent scanning procedures. However, 15 (controls = 6, CR = 9) were excluded from this analysis due to lesions visible on the T1- and T2-weighted scans (*N* = 6), poor image quality as a result of breathing motion, phase artifact, technical issues (*N* = 6), or incomplete scans from excessive involuntary movements despite sedation (*N* = 3).

### Image processing

Image preprocessing methods have been described previously and are only briefly summarized here (Bendlin et al., 2011; Willette et al., 2012b). T1-weighted images were manually skull-stripped, rotated, segmented, and normalized to 112RM-SL atlas space (McLaren et al., 2009) using Statistical Parametric Mapping software (SPM5, http://www.fil.ion.ucl.ac.uk/spm/software/spm5/) and using DARTEL (Ashburner, 2007). The segments were smoothed using a 4 mm FWHM Gaussian kernel. GM volumes were masked with binary transforms of prior probability maps. FA and MD maps were computed using DTIFIT in FSL (http://www.fmrib.ox.ac.uk/fsl), aligned to the B0 map, which was then aligned to the 112RM-SL atlas space in SPM, and smoothed using a 4 mm FWHM Gaussian kernel. FA analyses were masked using a binary WM mask, whereas MD analyses were masked using a combined GM and WM mask. All MD results are reported in the Appendix.

### Statistical analyses

Of the 22 animals (controls = 4, CR = 18) that completed the WCST, only 15 animals (controls = 3, CR = 12) had usable scans and were thus included in the statistical analyses. Due to the small number of controls that completed WCST task, the animals were pooled across groups for all analyses. For the motor analyses, the sample size included up to 27 animals (controls = 16, CR = 11) depending on the availability of a usable scan and successful completion of the respective level of motor task.

Multiple regression voxel-wise analyses were conducted in SPM8 using a general linear model. GM, FA, or MD map was used as dependent variables, and performance on the cognitive and motor tasks was used as the independent variable in separate models. To ensure a normal distribution, cognition and motor scores were Winsorized (Wilcox, 2012) to ±2 standard deviations of the mean. Given the limited sample size for WCST completion in controls, only age and global signal were used as covariates. For motor analyses, age, gender, dietary condition, and global signal variable were used as covariates. In a separate model, a motor performance by dietary condition interaction term was added to assess the extent to which CR affected the relationship between brain health and executive function. For each analysis, an omnibus *F*-contrast (*p* < 0.05, uncorrected) was computed and used to mask subsequent *t*-contrasts. The voxel level threshold was set at *p* < 0.005 (uncorrected). Cluster threshold was set to 100 contiguous voxels. Cluster locations were identified using standard rhesus macaque atlases (Schmahmann and Pandya, 2006; Saleem and Logothetis, 2007) and correspond to the Saleem–Logothetis atlas coordinates. Results are displayed on the 112RM-SL atlas template.

## Results

### Demographics

Table 1 shows the subject characteristics and performance times of the animals included in the brain-behavior correlations. The mean age and gender composition did not differ significantly between the two dietary conditions.

### Executive function, age, and CR

**Figure 1:**
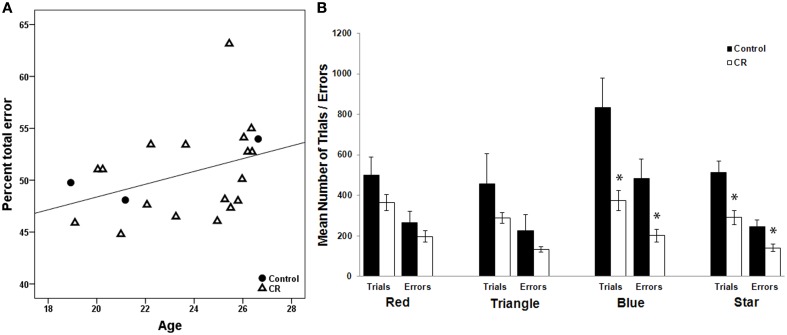
**(A)** There is a significant positive relationship between age and percentage of total errors to total trials on the WSCT across 4 control and calorie restricted (CR) animals (Spearman's coefficient = 0.498, *p* = 0.022). **(B)** The effect of CR on WCST performance. The trials needed to reach criteria and errors committed are depicted for each testing paradigm. ^*^Denotes a significant difference between the two dietary groups (*p* < 0.05).

There was a significant positive correlation between age and %TE among all animals that successfully completed WCST (Spearman's rho = 0.498, *p* = 0.022, Figure 1A). There was no significant relationship between %PE and age. Mann–Whitney U Test indicated that CR and control animals did not differ when initially learning to distinguish a specific color (“red”) or shape (“triangle”) as the correct stimulus, but CR animals spent significantly fewer trials as compared to controls mastering the task when a new class of color (“blue”) or shape (“star”) was introduced (*p* < 0.05, Figure 1B). The average response latencies did not differ significantly between the two groups (control = 2.92 ± 1.38 s, CR = 5.27 ± 3.62 s, *p* = 0.221). The mean percent perseverative and total errors for subjects included in the brain-behavior analyses are presented in Table 1.

#### Executive function and gray matter volume

**Figure 2:**
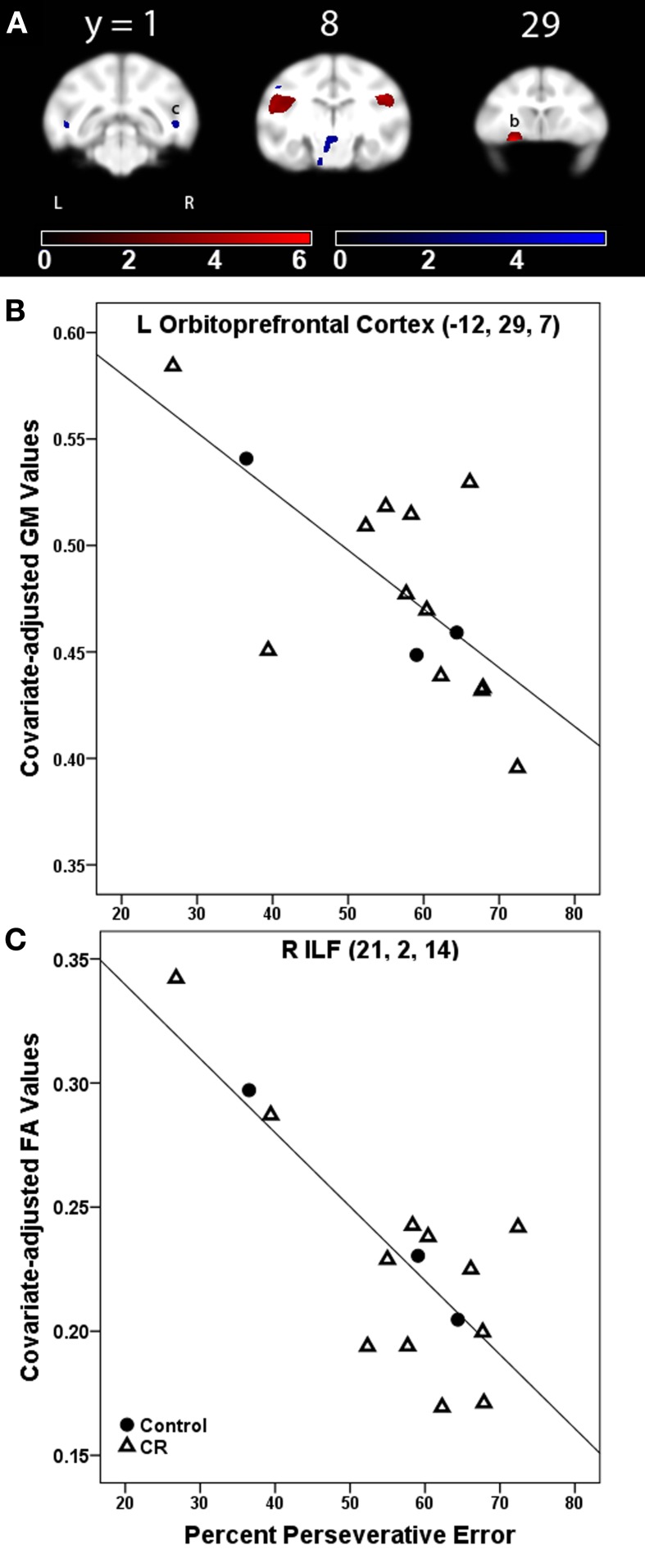
**(A)** Statistical parametric map of the *t*-contrast displayed on coronal slices showing regions where higher percent perseverative error was correlated to less gray matter (GM, red) and lower fractional anisotropy (FA, blue) values for all animals (controls and CR). All comparisons include age and global signal variable as covariates. The probability threshold for each *t*-contrast was *p* < 0.005 (uncorrected). The color bars represent the value of the *t*-statistic. The left side of the brain is on the left in the images. **(B)** Plot of the negative correlation between perseverative error and regional GM in left orbitoprefrontal cortex, labeled as (b) in **(A)**. **(C)** Plot of the correlation between higher perseverative error and lower FA at the right inferior longitudinal fasciculus [ILF, labeled as (c) in **(A)**].

%PE was related to less GM in bilateral secondary somatosensory areas, left orbital prefrontal cortex, left cerebellum, and right opercular area (Figure 2A, GM, red). Table 2 provides the *t-statistic*, cluster size, and 112RM-SL coordinates for the specific regions where higher %PE was associated with lower GM volume. This negative relationship in the orbitoprefrontal cortex is displayed in Figure 2B. There were no significant associations between %TE and GM after controlling for age.

**Table 2:** **Regions where perseverative error rate was significantly correlated with gray matter volume and fractional anisotropy in voxel-wise analyses**.

**Location**	***t*-statistic**	***x, y, z***	**Cluster size (voxels)**
**GRAY MATTER: NEGATIVE ASSOCIATION**			
L somatosensory area 2	6.26	−24, 12, 17	3791
	4.91	−17, 6, 26	−
L orbitoprefrontal cortex	6.25	−12, 29, 7	918
	3.3	−8, 22, 3	−
R parietal operculum	5.19	18, 8, 24	806
	3.24	24, 12, 20	−
L cerebellum	4.45	−16, −8, 4	199
L somatosensory areas 1−2	4.31	−6, 6, 33	434
**FRACTIONAL ANISOTROPY: NEGATIVE ASSOCIATION**			
R ILF	5.20	21, 2, 14	183
L CP	3.61	−4, 10, 4	1333
L ILF	5.17	−22, 2, 13	130
L ILF	3.78	−14, 6, 1	142

Notes: L, left; R, right; ILF, inferior longitudinal fasciculus; CP, cerebral peduncle.

#### Executive function and white matter fractional anisotropy

%PE was associated with lower FA in bilateral inferior longitudinal fasciculus (ILF) and left cerebral peduncle (Figure 2A, FA, blue). Table 2 indicates the *t*-statistic, cluster size, and 112RM-SL coordinates for the specific regions where higher %PE was associated with lower FA values. This negative association in the right ILF is shown in Figure 2C. There were no significant clusters where there was a negative association between %TE and FA.

### Motor function, age, and CR

There was a direct relationship between age and fine movement time on the straight rod task (Pearson correlation = 0.449, *p* = 0.021). The average reaction and coarse movement times for the mMAP are provided in Table 1 and did not vary significantly between the two dietary groups. We have previously reported that control animals are significantly slower in both learning and performing at proficiency the fine motor component of the most difficult task compared to CR animals; there was no significant group difference for the two easier levels of the task (Kastman et al., 2010).

#### Coarse movement time

***Gray matter***. There was a negative association between coarse movement time and GM volume across all subjects in bilateral visual areas, bilateral temporal cortex regions such as area TE and superior temporal sulcus, and left cerebellum. These results are shown in Table A2.

***Gray matter: coarse movement time × dietary group interaction***. GM regions where coarse movement time correlations showed significantly steeper slopes in controls as compared to CR animals included right temporal and visual cortex (Table A2).

***Fractional anisotropy***. Coarse movement time was negatively correlated to FA in bilateral ILF and left sagittal stratum (SS). These results are shown in Table A2.

***Fractional anisotropy: coarse movement time × dietary group interaction***. Coarse movement time correlations showed significantly steeper slopes in controls as compared to CR animals in the right SLF II and bilateral cerebral peduncles (Table A2).

#### Fine movement time

**Figure 3:**
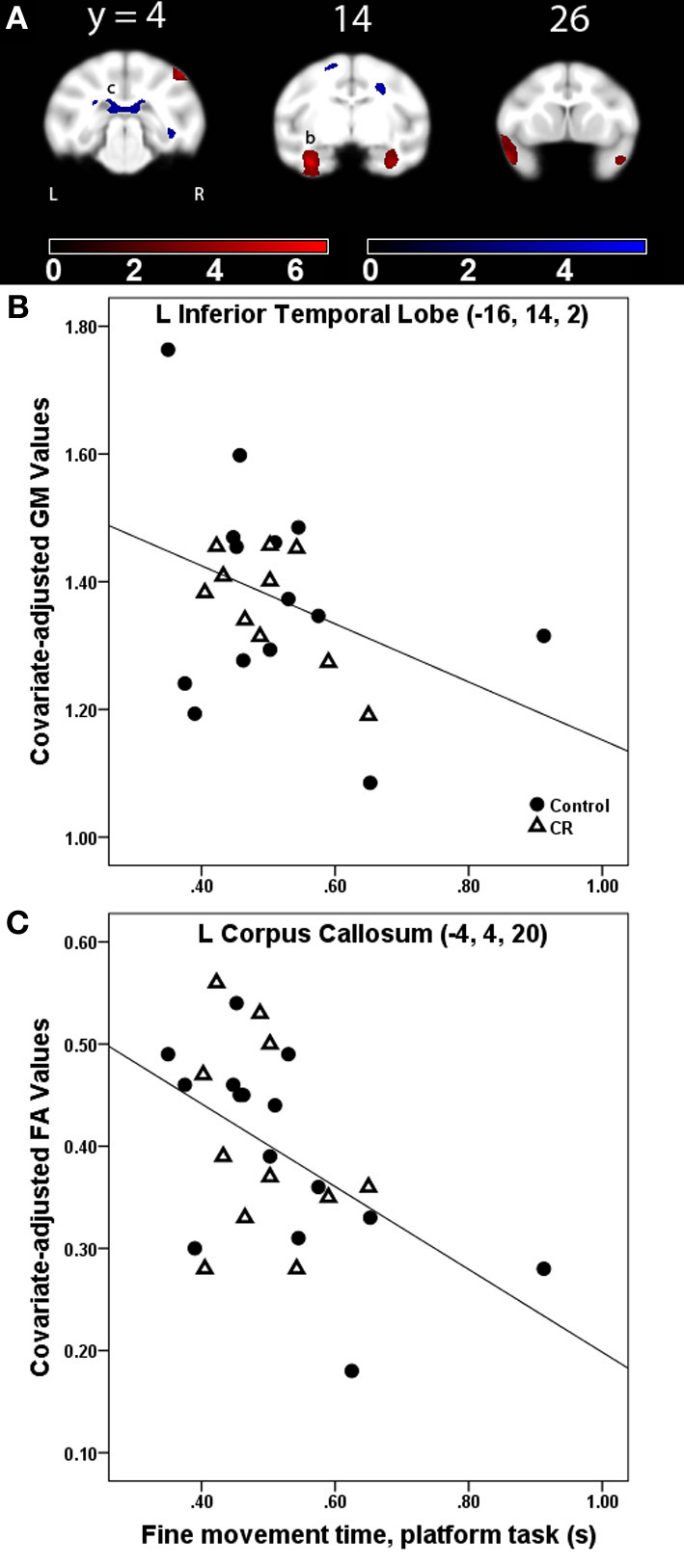
**(A)** Statistical parametric map of the *t*-contrast displayed on coronal slices showing regions where greater fine movement time (in seconds) on the platform task was correlated to less gray matter (GM, red) and lower fractional anisotropy (FA, blue) values for all animals (controls and CR). All comparisons include age, gender, dietary condition, and global signal variable as covariates. The probability threshold for each *t*-contrast was *p* < 0.005 (uncorrected). The color bars represent the value of the *t*-statistic. The left side of the brain is on the left in the images. **(B)** Plot of the negative correlation between fine movement time (in seconds) on the platform task and regional GM at the labeled location in the inferior temporal lobe as (b) in panel **(A)**. **(C)** Plot of the correlation between higher fine movement time (in seconds) on the platform task and lower FA at the left corpus callosum, labeled as (c) in **(A)**.

***Gray matter***. Fine movement time on the first level of mMAP task was related to less GM volume in bilateral inferotemporal cortex, right parietal operculum, right frontal area, left cerebellum, and left visual areas (Figure 3A, GM, red). Figure 3B depicts this negative relationship in the left inferior temporal cortex across all subjects. There was a negative correlation between fine movement time on the straight rod task and GM volume across all subjects in left visual areas, left cerebellum, and right inferotemporal cortex. For the curved rod task, regions showing a negative association between fine movement time and GM included left visual areas and cerebellum during the learning phase, and left posterior visual areas, right middle cerebellar peduncle, and cerebellum during the proficiency phase. Table 3 lists the respective *t*-statistic, cluster size, and 112RM-SL coordinates for fine movement time correlations of clusters that exceeded 500 voxels; a complete listing is provided in Table A3.

**Table 3:** **Regions where fine movement time was significantly correlated with gray matter volume in voxel-wise analyses across subjects and between dietary groups**.

**Location**	***t*-statistic**	***x, y, z***	**Cluster size**
**ACROSS SUBJECTS**			
***Platform task***			
L anterior TE, ventral	6.76	−16, 14, 2	4270
L granular part of the dorsal temporal pole	5.48	−24, 26, 1	1254
R parietal operculum	5.00	22, 3, 32	1320
R anterior TE, ventral	4.75	16, 14, −1	2049
R area TH of the parahippocampal cortex	4.42	10, 7, 4	−
R ventral temporal pole	4.29	20, 28, −2	556
R precentral opercular area	4.06	22, 29, 7	−
L cerebellum	4.08	−10, −10, 2	824
***Straight-rod task***			
L visual area V3/V4	4.92	−24, −5, 12	1641
L cerebellum	4.67	−14, −5, −1	540
L visual area V1	3.47	−14, −16, 20	649
R area TEO	3.32	28, 0, 10	510
***Curved-rod task, proficiency phase***			
L visual area V1	4.24	−10, −22, 21	1376
L visual area V2	3.15	−18, −18, 26	−
L cerebellum	3.67	−11, −8, 1	509
**FINE MOVEMENT TIME × DIETARY GROUP INTERACTION**			
***Straight-rod task***			
R putamen	3.39	8, 26, 8	510
L amygdala	3.28	−14, 18, 2	510
***Curved-rod task, acquisition phase***			
L hippocampus/parahippocampal cortex	4.68	−14, 7, 4	3378
L entorhinal cortex, caudal division	4.56	−9, 14, −3	−
R entorhinal cortex, caudal division	4.36	8, 15, 0	2521
R area TF of the parahippocampal cortex	4.35	14, 8, 3	−
L STG, rostral	4.09	−22, 23, 3	976
***Curved-rod task, proficiency phase***			
L frontal area 11, medial	6.45	−4, 42, 16	520
L precentral opercular area	4.29	−26, 26, 9	853
L entorhinal cortex, caudal division	4.28	−9, 11, −2	796

Notes: L, left; R, right; STG, superior temporal gyrus. Only clusters with 500 or more voxels are reported here; refer to Appendix for complete table.

**Figure 4:**
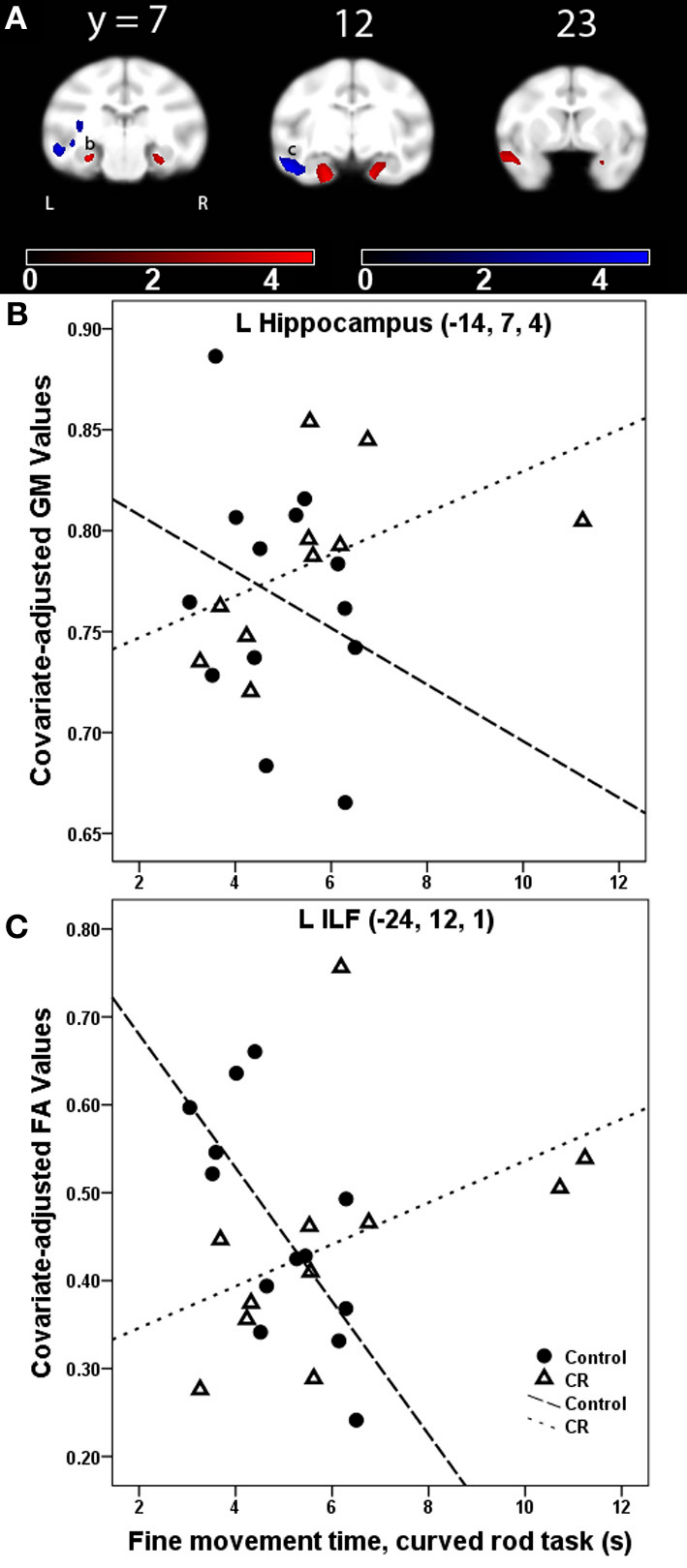
**(A)** Statistical parametric map of the *t*-contrast displayed on coronal slices showing regions where the correlation between fine movement time (in seconds) during the learning phase of the curved rod task and gray matter (GM, red) and lower fractional anisotropy (FA, blue) differs as a function of dietary group (controls vs. CR). All comparisons include age, gender, dietary condition, and global signal variable as covariates. The probability threshold for each *t*-contrast was *p* < 0.005 (uncorrected). The color bars represent the value of the *t*-statistic. The left side of the brain is on the left in the images. **(B)** Scatter plot of the correlation between fine movement time (in seconds) during the learning phase of the curved rod task and regional GM at the left hippocampus, denoted as (b) in **(A)**. **(C)** Plot of the correlation between fine movement time (in seconds) during the learning phase of the curved rod task and FA in the left inferior longitudinal fasciculus [ILF, location denoted as (c) in panel **(A)**].

***Gray matter: fine movement time × dietary group interaction***. For the platform task, regions showing a significantly steeper association between fine movement time and GM in controls than CR animals included anterior cingulate cortex, right putamen, and left middle temporal area. Regions showing fine movement correlations with significantly steeper slopes in the controls as compared to CR subjects during the straight rod task were left inferotemporal cortex, right cerebellum, right putamen, and left amygdala. During the learning phase of the curved rod task, these regions were present in bilateral temporal regions and right frontal area (Figure 4A, GM, red). Figure 4B portrays this difference in correlation between fine movement time during the learning phase of the curved rod task and regional GM in the left hippocampus as a function of dietary groups. During the proficiency phase of the curved rod task, significant clusters showing an interaction effect were present in the left frontal, right visual, and bilateral temporal areas. The respective *t*-statistic, cluster size, and 112RM-SL coordinates for fine movement time and diet group interaction effects of clusters that exceeded 500 voxels; a complete listing is provided in Table A4.

***Fractional anisotropy***. Fine movement time on the platform task was related to lower FA in several major WM tracts, including bilateral SS, left SLF I, bilateral SLF II, bilateral extreme capsule, right ILF, left cingulum bundle, corpus callosum, and right dorsal occipital bundle (Figure 3A, FA, blue). This negative relationship between higher fine movement time and lower FA in the left corpus callsoum is shown in Figure 3C. There was a negative correlation between fine movement time on the straight rod task and FA in the right fronto-occipital fasciculus, posterior limb of the right internal capsule (ICp), left SS, right SLF I, and left fornix. Fine movement time correlated positively to MD in right SLF III and left stria medullaris during the acquisition phase, and to right SLF III and posterior limb of the internal capsule during the proficiency phase of the curved rod task. Table 4 provides the respective *t*-statistic, cluster size, and 112RM-SL coordinates for fine movement correlations of clusters that exceeded 500 voxels; a complete listing is provided in Table A4.

**Table 4:** **Regions where fine movement time was significantly correlated with fractional anisotropy in voxel-wise analyses across subjects and between dietary groups**.

**Location**	***t*-statistic**	***x, y, z***	**Cluster size**
**ACROSS SUBJECTS**			
***Platform task***			
R SS/ILF	5.66	18, 0, 10	1362
R ILF	3.95	24, −2, 16	−
L SLF II	4.64	−10, 8, 24	742
R SLF II	4.44	11, 14, 27	545
L SLF I	4.44	−10, 16, 34	501
L CC	3.65	−4, 4, 20	1477
R ILF	3.62	12, −3, 21	−
R CC	3.31	6, 5, 22	−
***Straight-rod task***			
R ICp	4.02	8, 12, 21	675
R SLF I	3.42	4, 26, 31	574
R SLF I	3.03	2, 34, 29	−
**FINE MOVEMENT TIME × DIETARY GROUP INTERACTION**			
***Platform task***			
L thalamus	5.31	−4, 8, 14	2988
L ICa	3.00	−4, 16, 10	−
R ICp	2.93	7, 8, 18	−
R UF	3.70	6, 40, 18	643
R UF	3.15	2, 40, 11	−
***Straight-rod task***			
L SLF I/II	5.62	−10, 17, 34	1248
R ICa	4.58	8, 20, 14	2272
R EC	4.06	12, 12, 15	−
R SS	4.45	18, 0, 12	855
L ICa	4.12	−8, 20, 14	1820
L EC	3.40	−13, 12, 16	−
***Curved-rod task, acquisition phase***			
L ILF	4.81	−24, 12, 1	2352
L ILF	3.62	−19, 19, −4	−
L SS	3.91	−18, 5, 10	556
***Curved-rod task, proficiency phase***			
R SS	5.48	18, 2, 10	608
L ILF	5.36	−24, 8, 5	4135
L ILF	4.99	−18, 14, −1	−
R EmC	3.74	16, 12, 13	555
R SLF II	3.18	12, 12, 21	−

Notes: L, left; R, right; SS, sagittal stratum; ILF, inferior longitudinal fasciculus; SLF, superior longitudinal fasciculus; CC, corpus callosum; ICa, internal capsule, anterior limb; ICp, internal capsule, posterior limb; UF, uncinate fasciculus; EC, external capsule; EmC, extreme capsule; CP, cerebral peduncle. Only clusters with 500 or more voxels are reported here; refer to Appendix for complete table.

***Fractional anisotropy: fine movement time × dietary group interaction***. WM regions where fine movement time during the platform task had significantly steeper slopes for the CR animals than controls included portions of the left thalamus, left SLF I and II, bilateral IC, right cingulum bundle, right uncinate fasciculus, and right pontine bundle. For the straight rod task, regions that showed such an interaction were found in left SLF I/II, anterior limb of the internal capsule (ICa) bilaterally, right external capsule, bilateral SS, right cerebellum, and bilateral cerebral peduncles. Clusters that showed an interaction between fine movement time and dietary group were present in the left ILF, bilateral SS, right cerebral peduncle, and bilateral SLF II during the learning phase (Figure 4A, FA, blue), and in bilateral SS, left ILF, bilateral cerebral peduncles, right SLF II, right extreme capsule, and right ICa during the proficiency phase of the curved rod task. Figure 4C shows this interaction effect between fine movement time during the learning phase of the curved rod task and FA in the left ILF as a function of dietary groups. Table 4 presents the respective *t*-statistic, cluster size, and 112RM-SL coordinates for fine movement and diet group interaction effects of clusters that exceeded 500 voxels; a complete listing is provided in Table A4.

## Discussion

Our analyses demonstrate that aged macaques on long-term CR acquire the most difficult components of the WCST in fewer trials, and make fewer total and perseverative errors during testing. Performance on this task of executive function and on the mMAP motor task was associated with GM volume and WM tissue integrity in several brain regions. Specifically for the cognitive task, perseverative errors on the WCST were related to less GM volume in frontal and parietal cortices, and decreased FA in the ILF. Likewise, increased time to perform various components of the motor task was associated with less GM and increased MD in cortical and subcortical areas throughout the brain, and low FA and high MD values in several major association fiber bundles. These data are largely in agreement with similar studies done in humans, where age-related brain changes are related to age-associated cognitive changes (Raz et al., 1998; Gunning-Dixon and Raz, 2000, 2003; Bendlin et al., 2010). Interestingly, the relationship between brain imaging indices and behavioral measures were significantly greater in magnitude among the controls such that worse performance (higher error rate) was associated with less regional GM volume, low FA, and high MD values. Conversely, in CR animals, these brain-behavior relationships were attenuated, possibly due to the protective effect of CR on both brain aging and cognitive function.

### Executive function, age, and CR

Performance on the WCST, a test of executive function task involving conceptual shifting, has been shown to decline as a function of age, both in humans and rhesus macaques (Rhodes, 2004; Makris et al., 2007). Makris et al. (2007) demonstrated significant age-related decline in WM integrity in the frontal fibers such as SLF II, cingulum bundle, and anterior corpus callosum, which in turn was directly correlated to performance on a test of executive function, but not recognition memory, in rhesus monkeys. Similar to previous studies, we found a direct relationship between age and total errors on the WCST, and interestingly, poor performance on the WCST was associated with low GM volume, with several clusters in frontal and parietal areas showing a relationship with more perseverative errors on the WCST task. Several lesion studies in monkeys have shown that performance on cognitive tests is severely impaired with frontal lobe dysfunction (Mishkin and Manning, 1978; Passingham, 1985a,b). Specifically, rhesus monkeys with lesions of the dorsolateral prefrontal cortex are impaired in efficient creation and manipulation of abstract rules in a modified WCST task (Moore et al., 2009). While dorsolateral prefrontal cortex is important for some aspects of rule-guided behavior, monkeys with lesions in this area do not appear to perseverate in their use of particular rules in the task, suggesting the involvement of other prefrontal areas in different aspects of the executive function and rule-guided behavior (Baxter, 2009). In marmosets, orbitofrontal cortex lesion is associated with an impaired ability to reverse a stimulus-reward association within a perceptual dimension (Dias et al., 1997). The orbitofrontal cortex in rhesus monkeys is also involved in associative learning, and processes reward and affect information (Meunier et al., 1997; Wallis and Miller, 2003). In humans, fMRI studies using normal controls, patients with frontal brain lesions, and patients with psychiatric illnesses related to frontal lobe dysfunction, such as schizophrenia, have also demonstrated a correlation between frontal lobe activity and WCST performance (Rogers et al., 2000; Monchi et al., 2001; Bonilha et al., 2008; Mukhopadhyay et al., 2008). In addition, it is important to note the involvement of cerebellar areas in the present cognitive task, as it is likely that the contribution of other cortical and cerebellar regions is critical for optimal functioning in this cognitive domain (Berman et al., 1995; Moore et al., 2009). For instance, WCST performance is consistently related to the cerebellum and anterior cingulate cortex in humans (Schmahmann and Sherman, 1998; Rogers et al., 2000; Wilmsmeier et al., 2010; Pedersen et al., 2012). Greater perseveration was also related to low FA in the inferior longitudinal fasciculus, which links the temporal and occipital lobes and is involved in object recognition, discrimination, and memory (Schmahmann et al., 2007). Many control monkeys could not be shaped to complete the WCST, but given no group difference in response latencies among the animals who did complete the task, poorer performance of the control group was likely not due to motivational factors. Consequently, we were unable to test for an interaction effect of dietary group and behavior. However, we did observe that CR animals showed high executive function, evidenced by their ability to more quickly acquire an extra-dimensional set shift between color shape and change.

### Motor function, age, and CR

Regarding motor function, we found that low regional GM volume, low FA, and high MD were associated with slower performance on the motor task in cortical, subcortical, and cerebellar areas, as well as long association bundles, which is similar to previously published studies. We did not find any correlations with GM volume in motor or premotor cortices, yet there were significant clusters in subcortical areas such as putamen and thalamus, and fiber bundles such as internal capsule, cerebral peduncle, and SLF I, which are well known to regulate motor behavior (Doyon et al., 2003; Makris et al., 2005). Indeed, motor deficits are commonly described in neurodegenerative states involving these regions (e.g., Parkinson's disease). The basal ganglia and cerebellum are specifically important for motor sequence learning and adaptation, whereas the hippocampus and medial temporal lobe, along with the striatum, are critical for both motor sequence learning and consolidation (Doyon et al., 2003; Lacreuse et al., 2006; Albouy et al., 2008). The role of frontal, parietal, and temporal areas and deep cerebellar nuclei in reaching and grasping movements has been reported in prior studies in monkeys using electrophysiological, anatomic connectivity, and positron emission tomography methods (Murata et al., 2000; Gregoriou and Savaki, 2003; Nishimura et al., 2007; Borra et al., 2008). It is likely that these non-motor cortical regions and related fiber bundles process somatosensory and visual information about the shape, orientation, and size of the object, and relevant spatial and directional information in order to efficiently reach out with the arm and grasp the food reward from the inner chamber. In addition to fine motor coordination and dexterity, there is also a cognitive component to this task, as the monkey must develop a strategy for efficient retrieval of the food reward, especially when engaged in the more difficult task levels (Moore et al., 2010). That several correlations are apparent for the easier task levels than the curved rod component might be related to the novelty of the mMAP apparatus during early test stages, and such processing may cease to be significant as the monkey becomes acclimated to testing apparatus.

We also observed several brain regions where CR seemed to lessen the age-related brain-behavior decline. In contrast to across-subject regression analyses, these interaction effects were more apparent in the more complex levels of the task, which are likely related to significantly improved performance of CR animals on the difficult component as compared to controls (Kastman et al., 2010). For instance, slower performance during the learning phase of the curved rod level was more strongly correlated to less GM volume in the hippocampal and parahippocampal area in controls as compared to CR animals (Figure 4), suggesting that a CR diet may ameliorate the effect of unit change in GM volume on motor behavior and positively influence task performance. This result concurs with several previous reports indicating that CR has a beneficial effect on delaying the aging process, in non-primate animal models as well as in primates (Heilbronn and Ravussin, 2003; Kemnitz, 2011). Previous studies from our group have shown that GM volume is preserved in midcingulate cortex, bilateral lateral temporal cortex, and right dorsolateral frontal cortex, and WM integrity in the fronto-occipital fasciculus, SLF, external capsule, and brainstem as a function of CR in monkeys (Colman et al., 2009; Bendlin et al., 2011). Superior performance of CR animals on tasks of fine motor movement is also related to improved glucoregulatory profiles in these animals (Willette et al., 2012a). Given that these behavioral correlations extend across imaging modalities that examine volumetric as well as tissue density changes, it likely that CR mediates these effects via several mutually non-exclusive mechanisms, such as decreasing oxidative stress and inflammation in the brain, ultimately resulting in increased neuronal survival, synaptic plasticity and improved microstructural health (Mattson, 2000; Heilbronn and Ravussin, 2003; Weindruch, 2003; Ingram et al., 2007). For instance, we have previously shown that CR is associated with lower levels of proinflammatory cytokines like IL-6 (Willette et al., 2010). Maswood and colleagues (Maswood et al., 2004) showed that adult male rhesus monkeys on a 30% short-term (6 months) CR diet perform better on the most difficult component of the mMAP motor task 6 weeks after a neurotoxic insult compared to controls (not statistically significant, however, due to small sample size). Using postmortem neurochemical analyses, they also demonstrated that these CR animals had higher levels of neurotrophic factors. While it is not feasible to directly process histological tissue in the current study due to its ongoing nature, it is likely that the improved performance that CR animals exhibit on manual dexterity tasks is due to the overall salubrious effect of CR on brain health. Such samples will be collected upon death to evaluate brain levels of neurotrophic factors, oxidative metabolites, and inflammatory markers to understand potential mechanisms of CR.

### Limitations

There are some limitations that should be noted. First, the age range of the subjects studied in these analyses is limited. The subjects are part of a larger cohort of monkeys, most of which are middle-aged to aged at the time they underwent imaging and behavioral testing, maintained longitudinally to study the effect of CR. The current study therefore only permits interpretations of age effects over a short range. Secondly, the current study assessed executive performance in these animals using one task. The findings may still possibly be confounded by motivational issues, since few control animals could be shaped to complete the task in its entirety. Nonetheless, given no significant group difference in response latencies in the animals that did complete the task, that the few control values in the executive function task were qualitatively in the same general range as that of the CR animals, and that only animals that fully completed the task were included in the analyses, these effects likely reflect normal aging changes. Third, the small sample size for the cognition analyses made it difficult to separate the protective effects of CR from changes associated with normal brain aging. Larger sample sizes in general allow for a more conservative voxel threshold than used in the current study and would provide more power to examine underlying interaction effects. The current cross-sectional study design also did not permit inferences of causation. Since several of the animals in this study are still alive, longitudinal analyses can more thoroughly inform us of the effects of normal brain aging and any protective effects of CR in mitigating the aging processes. Finally, although regional GM and DTI-derived indices are clearly correlated to cognitive and motor performance, the underlying microscopic alterations and mechanisms mediating these aging changes remain to be investigated and will be the topic of future investigations.

### Conflict of interest statement

The authors declare that the research was conducted in the absence of any commercial or financial relationships that could be construed as a potential conflict of interest.

## Appendix

### Results

#### Executive function and mean diffusivity

No clusters exceeded the threshold for a significant positive association between %PE and MD. There was a positive association between %TE and MD in right area TPO of the temporal sulcus, bilateral auditory cortex, hypothalamus, right anterior commissure, and left ventromedial prefrontal cortex (Table A1).

**Table A1:** **Regions where total error rate was significantly correlated with mean diffusivity in voxel-wise analyses**.

**Location**	***t*-statistic**	***x, y, z***	**Cluster size (voxels)**
**MD: POSITIVE ASSOCIATION**			
R area TPO	5.81	22, 1, 24	350
R auditory cortex	5.28	26, 10, 20	236
L auditory cortex	4.58	−26, 8, 22	148
R anterior commissure	4.18	11, 19, 8	326
R putamen	3.12	15, 12, 10	−
Hypothalamus	3.77	0, 18, 10	355
L ventromedial prefrontal area 14c	3.69	−3, 26, 8	150

Notes: R, right; L, left; SLF, superior longitudinal fasciculus.

#### Coarse movement time

***Mean diffusivity***. Regions where coarse movement time correlations were positively related to MD across all subjects included cerebellum and posterior visual areas bilaterally. These results are shown in Table A2.

**Table A2:** **Regions where coarse movement time was significantly correlated with gray matter volume, fractional anisotropy, and mean diffusivity in voxel-wise analyses across subjects and between dietary groups**.

**Location**	***t*-statistic**	***x, y, z***	**Cluster size**
**GM: NEGATIVE ASSOCIATION**
L visual area V4	6.03	−23, −6, 11	1969
L anterior TE, ventral	4.58	−16, 16, −6	1230
R anterior TE, ventral	4.19	15, 20, −4	2291
R sts part of the temporal pole	3.49	19, 27, −4	−
L cerebellum	4.07	−14, −9, 0	651
R visual area V4	4.02	23, 2, 8	562
L sts part of the temporal pole	3.65	−22, 26, −2	384
L visual area V1	3.42	−17, −14, 20	384
**GM: COARSE MOVEMENT TIME × DIETARY GROUP INTERACTION**			
R rostromedial belt region, auditory cortex	5.21	21, 11, 12	664
R visual area V2	3.72	28, −2, 18	870
**FA: NEGATIVE ASSOCIATION**			
R ILF	4.91	18, −10, 10	760
R SS	3.54	17, 0, 10	−
L ILF	3.77	−18, −12, 10	206
L SS	3.67	−17, 2, 11	282
**FA: COARSE MOVEMENT TIME × DIETARY GROUP INTERACTION**			
R SLF II	4.13	9, 24, 25	285
R CP	3.49	4, 10, −2	309
L CP	3.44	−4, 10, −2	−
**MD: POSITIVE ASSOCIATION**			
R cerebellum	5.40	20, −8, 8	383
R SS	2.93	18, −4, 16	−
L cerebellum	3.92	−18, −10, 9	183
L lateral occipital parietal area	3.88	−10, −10, 34	306
R visual area V1	3.70	14, −16, 22	284
R visual area V3A	3.30	10, −8, 26	218

Notes: L, left; R, right; sts, superior temporal sulcus; ILF, inferior longitudinal fasciculus; SS, sagittal stratum; SLF, superior longitudinal fasciculus; CP, cerebral peduncle.

**Table A3:** **Regions where fine movement time was significantly correlated with gray matter volume in voxel-wise analyses across subjects and between dietary groups**.

**Location**	***t*-statistic**	***x, y, z***	**Cluster size**
**ACROSS SUBJECTS**			
***Platform task***			
L anterior TE, ventral	6.76	−16, 14, 2	4270
L granular part of the dorsal temporal pole	5.48	−24, 26, 1	1254
R parietal operculum	5.00	22, 3, 32	1320
R anterior TE, ventral	4.75	16, 14, −1	2049
R area TH of the parahippocampal cortex	4.42	10, 7, 4	−
R ventral temporal pole	4.29	20, 28, −2	556
R precentral opercular area	4.06	22, 29, 7	−
R frontal area 13a	4.15	4, 32, 8	211
L cerebellum	4.08	−10, −10, 2	824
L visual area 7a	3.72	−15, −2, 36	342
L dorsal prelunate area/visual area V4	3.22	−12, −9, 36	154
***Straight-rod task***			
L visual area V3/V4	4.92	−24, −5, 12	1641
L cerebellum	4.67	−14, −5, −1	540
L visual area V1	3.47	−14, −16, 20	649
R area TEO	3.32	28, 0, 10	510
***Curved-rod task, acquisition phase***			
L visual area V1	4.04	−4, −16, 30	442
L cerebellum	3.56	−12, −4, −2	165
L visual area V1	3.38	−14, −16, 24	288
L visual area V1	3.07	−20, −15, 28	139
***Curved-rod task, proficiency phase***			
L visual area V1	4.24	−10, −22, 21	1376
L visual area V2	3.15	−18, −18, 26	−
R middle cerebellar peduncle	4.06	13, 2, 2	303
L cerebellum	3.67	−11, −8, 1	509
**FINE MOVEMENT TIME × DIETARY GROUP INTERACTION**			
***Platform task***			
Anterior cingulate cortex, area 24b	3.45	0, 36, 22	305
R putamen	3.32	16, 14, 17	112
L middle temporal area	3.27	−20, −3, 27	143
***Straight-rod task***			
L area TF of the parahippomcapal cortex	4.81	−14, 6, 3	191
L area TE, ventral subregion of posterior area	3.80	−18, 9, −4	312
R cerebellum	3.54	14, −6, 0	330
R putamen	3.39	8, 26, 8	510
L amygdala	3.28	−14, 18, 2	510
***Curved-rod task, acquisition phase***			
L hippocampus/parahippocampal cortex	4.68	−14, 7, 4	3378
L entorhinal cortex, caudal division	4.56	−9, 14, −3	−
L frontal area 11, medial	4.60	−4, 44, 16	431
R entorhinal cortex, caudal division	4.36	8, 15, 0	2521
R area TF of the parahippocampal cortex	4.35	14, 8, 3	−
L rostral region of the superior temporal gyrus	4.09	−22, 23, 3	976
*Curved-rod task, proficiency phase*			
L frontal area 11, medial	6.45	−4, 42, 16	520
R visual area V1	5.39	22, −18, 12	381
R precentral opercular area	5.09	22, 27, 8	227
R entorhinal cortex	4.37	7, 11, −2	257
L precentral opercular area	4.29	−26, 26, 9	853
L entorhinal cortex, caudal division	4.28	−9, 11, −2	796

Notes: L, left; R, right.

**Table A4:** **Regions where fine movement time was significantly correlated with fractional anisotropy in voxel-wise analyses across subjects and between dietary groups**.

**Location**	***t*-statistic**	***x, y, z***	**Cluster size**
**ACROSS SUBJECTS**			
***Platform task***			
R SS/ILF	5.66	18, 0, 10	1362
R ILF	3.95	24, −2, 16	−
L SLF II	4.64	−10, 8, 24	742
R EmC	4.52	14, 10, 14	362
L SS	4.46	−18, −1, 10	394
R SLF II	4.44	11, 14, 27	545
L SLF I	4.44	−10, 16, 34	501
L EmC	3.82	−16, 8, 14	415
L CC	3.65	−4, 4, 20	1477
R ILF	3.62	12, −3, 21	−
R CC	3.31	6, 5, 22	−
L SLF II	3.51	−13, 18, 22	272
L CB	3.44	−12, 2, 10	163
L SLF I	3.28	−8, 26, 31	110
R dOB	3.27	7, −16, 27	112
***Straight-rod task***			
R FOF	4.16	9, 19, 25	178
R ICp	4.02	8, 12, 21	675
L SS	3.98	−16, 2, 14	314
R ILF	3.55	14, −7, 11	390
R SLF I	3.42	4, 26, 31	574
R SLF I	3.03	2, 34, 29	−
L fornix	3.25	−6, 8, 20	111
***Curved-rod task, acquisition phase***			
R SLF III	4.28	14, −2, 30	211
L stria medullaris	3.37	−2, 10, 16	269
***Curved-rod task, proficiency phase***			
R SLF III	3.67	16, −2, 29	159
R ICp	3.59	9, 14, 21	253
**FINE MOVEMENT TIME × DIETARY GROUP INTERACTION**			
***Platform task***			
L thalamus	5.31	−4, 8, 14	2988
L ICa	3.00	−4, 16, 10	−
R ICp	2.93	7, 8, 18	−
L SLF II	4.64	−16, 30, 26	211
L SLF I	4.32	−2, 16, 36	155
L ICa	4.26	−10, 26, 15	262
R CB	3.84	12, 8, 2	284
R UF	3.70	6, 40, 18	643
R UF	3.15	2, 40, 11	−
R PB	3.63	6, 8, −2	104
R ICp	3.37	10, 7, 14	148
***Straight-rod task***			
L SLF I/II	5.62	−10, 17, 34	1248
R ICa	4.58	8, 20, 14	2272
R EC	4.06	12, 12, 15	−
R SS	4.45	18, 0, 12	855
L SS	4.21	−18, 4, 11	356
L ICa	4.12	−8, 20, 14	1820
L EC	3.40	−13, 12, 16	−
L cerebellum	3.88	−4, −2, 4	299
R middle cerebellar peduncle	3.77	6, 4, 0	467
R cerebellum	3.53	8, −7, 4	123
R CP	3.39	1, 7, 6	111
L CP/PB	3.35	−6, 8, 1	126
***Curved-rod task, acquisition phase***			
L ILF	4.81	−24, 12, 1	2352
L ILF	3.62	−19, 19, −4	−
R SS	4.16	18, 4, 10	424
R CP	4.15	8, 12, 4	142
L SS	3.91	−18, 5, 10	556
L SLF II	3.60	−15, 1, 31	156
R SLF II	3.52	10, 15, 27	113
***Curved-rod task, proficiency phase***			
R SS	5.48	18, 2, 10	608
L ILF	5.36	−24, 8, 5	4135
L ILF	4.99	−18, 14, −1	−
R CP	5.23	5, 8, 0	380
R SLF II	4.52	11, 16, 28	478
R ILF	3.83	18, 18, −1	414
L SS	3.80	−16, −5, 20	209
R EmC	3.74	16, 12, 13	555
R SLF II	3.18	12, 12, 21	−
L CP	3.46	−8, 12, 4	115
R ICa	3.27	9, 18, 14	202

Notes: L, left; R, right; SS, sagittal stratum; ILF, inferior longitudinal fasciculus; SLF, superior longitudinal fasciculus; EmC, extreme capsule; CC, corpus callosum; CB, cingulum bundle; dOB, dorsal occipital bundle; ICa, internal capsule, anterior limb; ICp, internal capsule, posterior limb; UF, uncinate fasciculus; PB, pontine bundle; FOF, fronto-occipital fasciculus; EC, external capsule; CP, cerebral peduncle.

***Mean diffusivity: coarse movement time × dietary group interaction***. There were no regions that showed an interaction effect between coarse movement time and dietary group.

#### Fine movement time

***Mean diffusivity***. Fine movement time on the platform task was positively related to MD in bilateral frontal areas, right posterior primary visual cortex, left temporal cortex, and left fronto-occipital fasciculus. Regions that showed a positive correlation between MD and fine movement time during the straight rod task included bilateral ILF, portions of right thalamus, and left visual areas. For the curved rod task, regions that showed a positive relationship between MD and fine movement time included bilateral thalamus and right primary visual cortex during the learning phase, and bilateral visual cortices during the proficiency phase. Table A5 provides the respective *t*-statistic, cluster size, and 112RM-SL coordinates for fine movement time correlations with MD.

**Table A5:** **Regions where fine movement time was significantly correlated with mean diffusivity in voxel-wise analyses across subjects and between dietary groups**.

**Location**	***t*-statistic**	***x, y, z***	**Cluster size**
**ACROSS SUBJECTS**			
***Platform task***			
L frontal area 11l	4.41	−12, 44, 16	227
R visual area V1	4.16	20, −18, 18	1817
	3.43	28, −13, 14	−
L area TEa (sts dorsal bank)	3.95	−22, 14, 4	758
R frontal area 11l	3.91	10, 45, 16	562
L frontal area 12o	3.87	−19, 31, 6	103
L FOF	3.08	−8, 24, 29	181
***Straight-rod task***			
R ILF	3.84	20, −2, 12	238
R thalamus	3.63	4, 6, 18	150
L lateral occipital parietal area	3.54	−12, −10, 34	404
L visual area V2	3.41	−20, −10, 28	218
L ILF	3.37	−18, 2, 15	571
	3.13	−13, −2, 24	−
L dOB	3.17	−10, −10, 27	103
***Curved-rod task, acquisition phase***			
R thalamus	4.11	6, 7, 20	304
L thalamus	3.61	−8, 5, 18	337
R visual area V1	3.43	14, −18, 22	141
R visual area V1	5.98	14, −17, 22	812
L visual area V1	3.72	−12, −19, 22	267
**FINE MOVEMENT TIME × DIETARY GROUP INTERACTION**			
***Platform task***			
R cerebellum	4.85	20, −11, 6	1348
R CP	4.74	5, 10, −2	11824
L CP	4.61	−6, 10, −3	−
R SB/parahippocampal area	4.16	14, 12, 0	−
R frontal area 46v	4.57	−18, 35, 24	399
L STG, rostral	4.18	−26, 22, 2	1758
L STG, rostral	4.01	−28, 16, 10	−
L ICa	4.08	−4, 17, 13	2638
L STG, caudal	4.04	−30, 8, 16	172
L cerebellum	4.02	−12, −20, 8	275
R frontal area 13l	4.01	14, 36, 14	906
R intermediate agranular insula area	3.56	14, 30, 8	−
L dysgranular part of the dorsal temporal pole	3.92	−14, 26, 5	358
L EmC	3.86	−14, 6, 20	560
L SLF II	3.02	−10, 1, 26	−
L dOB	3.79	−19, −6, 28	199
R agranular frontal area F5	3.66	26, 26, 10	1540
R anterior lateral belt region, auditory cortex	3.27	26, 16, 14	−
R sts part of the temporal pole	3.26	18, 26, −2	−
L visual area V2	3.63	−22, −14, 13	113
L agranular frontal area F2	3.52	−5, 24, 38	331
L visual area V2	3.48	−12, −6, 16	110
L putamen	3.40	−16, 20, 8	192
L visual area V1	3.35	−18, −20, 22	109
L brainstem	3.33	−4, −9, −4	198
R EmC	3.28	13, 6, 20	292
L cerebellum	3.19	−17, −12, 6	153
L anterior TE, dorsal subregion	3.11	−20, 18, −3	317
***Straight-rod task***			
L SLF II	5.47	−10, 25, 30	4434
L SLF I	4.37	−10, 16, 32	−
L thalamus	4.15	−2, 14, 17	506
R visual area V1	3.63	26, −8, 20	444
R visual area V2	3.43	30, −6, 14	−
L SLF II	3.61	−16, 16, 23	272
L area TEa (sts ventral bank)	3.47	−22, 9, 4	179
L cerebellum	3.36	−10, −15, −2	105
R visual area V1	3.18	22, −12, 28	173
***Curved-rod task, acquisition phase***			
R ILF	5.59	24, 11, 0	2090
L ILF	4.68	−22, 10, 4	1301
R visual area V2	4.48	28, −6, 14	272
L visual area V1	4.34	−12, −24, 24	872
R visual area V1	3.82	10, −26, 23	335
***Curved-rod task, proficiency phase***			
L ILF	7.59	−23, 9, 4	9720
	4.78	−16, 20, 5	−
	4.35	−13, 14, 0	−
R ILF	5.98	22, 10, 0	8342
	5.43	28, 18, 6	−
	4.73	14, 20, 6	−
R visual V1	5.31	19, −21, 17	6266
	4.53	22, −14, 27	−
	3.99	10, −24, 24	−
R brainstem	5.05	6, −2, −5	1019
	3.68	−4, −2, −6	−
R visual area 7a	4.30	18, 0, 36	838
R visual area V4	3.65	26, −4, 28	−
R visual area V2	3.17	18, −9, 34	−
L visual area V1	4.17	−18, −21, 20	525
L SLF I	4.06	−8, 19, 32	667
R SLF II	3.61	8, 22, 26	574
L SLF II	3.45	−12, 12, 26	240

Notes: L, left; R, right; sts, superior temporal sulcus; FOF, fronto-occipital fasciculus; ILF, inferior longitudinal fasciculus; dOB, dorsal occipital bundle; CP, cerebral peduncle; SB, striatum bundle; STG, superior temporal gyrus; ICa, internal capsule, anterior limb; EmC, extreme capsule; SLF, superior longitudinal fasciculus.

***Mean diffusivity: fine movement time × dietary group interaction***. Regions where fine movement time during the platform task was associated with stronger correlations in the controls as compared to CR included the cerebellum, areas from the temporal frontal, and visual cortex, and WM tracts such as the cerebral peduncles, and left internal and extreme capsules. For the straight rod task, clusters showing an interaction effect were present in left SLF, left thalamus, left temporal regions, and right visual regions. For the curved rod task, voxel clusters showing an interaction effect between fine movement time and dietary group were seen in bilateral ILF and visual regions during the learning phase, and in bilateral ILF, SLF, primary visual cortex, and right brainstem during the proficiency phase. The respective *t*-statistic, cluster size, and 112RM-SL coordinates for fine movement and diet group interaction effects are presented in Table A5.
